# Asymmetric Stereo High Dynamic Range Imaging with Smartphone Cameras

**DOI:** 10.3390/s24185876

**Published:** 2024-09-10

**Authors:** Finn Russell, William J. B. Midgley

**Affiliations:** School of Mechanical and Manufacturing Engineering, UNSW Sydney, Sydney, NSW 2052, Australia

**Keywords:** high dynamic range, stereo imaging, deep learning, asymmetric imaging

## Abstract

Stereo high dynamic range imaging (SHDRI) offers a more temporally stable solution to high dynamic range (HDR) imaging from low dynamic range input images compared to bracketing and removes the loss of accuracy that single-image HDR solutions offer. However, few solutions currently exist that take advantage of the different (asymmetric) lenses, commonly found on modern smartphones, to achieve SHDRI. This paper presents a method that achieves single-shot asymmetric HDR fusion via a reference-based deep learning approach. Results demonstrate a system that is more robust to aperture and image signal processing pipeline differences than existing solutions.

## 1. Introduction

Conventional digital camera systems rely on sensors that capture only a limited range of intensity values. In high dynamic range (HDR) scenarios, the camera cannot capture sufficient information, leading to a loss of detail in saturated (over-exposed) or under-exposed areas. These limitations have encouraged extensive research into converting input LDR (low dynamic range) images into HDR output images [1]. In stereo HDR (SHDR) scenarios, these input images are shot on separate cameras, which inform HDR image generation via a process of exposure estimation, image alignment and fusion [2].

Despite increasing interest in the field, existing SHDR algorithms and models do not accommodate aperture and image signal processor (ISP) differences inherent to smartphone camera systems with multiple lenses. The majority of currently proposed methods assume the images have been taken on identical cameras; as such, differences in field of view (FoV), resolution and general camera properties have not been considered. To the knowledge of the authors, the only existing solution to account for these differences relies on multiple images per camera-lens, increasing temporal instability [3].

The increasing prevalence of multiple lenses on smartphone systems has encouraged research into reference-based image processing, where a ‘reference’ image informs operations performed on a ‘query’ image. Existing works have achieved reference-based smartphone image processing for super resolution [4,5] and focus synthesis [6]. Deep learning (DL) has accelerated the effectiveness and efficiency of reference-based solutions, with many processes reliant on localised reference-based image matching techniques such as optical flow [7,8].

Despite growing interest in the field, existing HDR algorithms and DL models face the following limitations:

1. Difficulties with aligning images from asymmetric lenses at different exposures.
2. Difficulties with temporal instability when capturing multiple images at different exposures from a single lens for HDR.

This paper will address these challenges to develop an SHDRI pipeline that accounts for asymmetry in ISP, aperture and resolution called ASHDRI (asymmetric stereo high dynamic range imaging) that outperforms existing SHDRI implementations. Specifically, this new pipeline achieves the following:

1. Aligns differently exposed images from different lenses, each with different properties.
2. Fuses the aligned LDR images into a single HDR image.
3. Is accurate, robust and temporally stable, using only two heterogeneous images as inputs.

This paper is laid out as follows: First, in Section 2, a discussion of existing works is presented. Section 3 introduces the novel ASDHRI pipeline and explains how it was tested against existing HDR implementations. Section 4 provides results from the new ASHDRI pipeline and some discussion on them. Finally, some conclusions are drawn and future work suggested in Section 5. The code for this project has been made open-access and is available on GitHub (https://github.com/Frussell556/ASHDRI, accessed on 26 August 2024).

## 2. Existing Works

Few works exist in the asymmetric stereo or multi-view HDR space. However, methods employed by this paper have been inspired by a few key works.

### 2.1. Multi-Modal Image Fusion

Xu et al. developed a deep learning method called RFNet for image fusion of multi-modal images with asymmetry that uses a coarse then fine two-pass image registration technique for image alignment [9]. This work achieved good results for combining multi-modal—in this case, near-infrared (NIR) and visible-light—images. However, the work did not investigate applying this method to visible-light images with large asymmetries. Furthermore, the work focused on the alignment task, assuming that the two images are well-exposed, unlike work in HDR imaging where images can have a range of exposures.

### 2.2. Depth Estimation

Chari et al. [3] designed a dual SHDR and depth estimation system using asymmetric lenses. They first estimated the scene radiance, then captured a stack of differently exposed images from each camera before iteratively estimating the disparity between stacks and the inverse camera response function (iCRF). As LDR images are taken at consistent exposure values (EVs) across lenses, their alignment process is achieved per image pair. The LDR images are then fused together and a final disparity estimation is generated. Whilst their work does successfully produce a well-resolved HDR image, it fails in relation to temporal stability. Their method makes extensive use of exposure stacks for both HDR fusion and image warping/alignment, resulting in temporal artefacts being propagated across the entire imaging pipeline. Additionally, their method makes no use of DL methods, limiting its robustness and efficacy.

### 2.3. Optical Flow

Optical flow-based alignment warps images on a dense, per-pixel basis via flow vector fields. Convolutional neural networks (CNNs) such as FlowNet have seen extensive use in optical flow alignment problems due to their increased accuracy, efficiency and robustness in solving flow-warping problems. Peng et al. [10] and Prabhakar et al. [11] both proposed replacing the conventional alignment process with optical flow CNNs. As optical flow’s estimation assumes that the inputted images are of the same exposure levels, all images are matched to a single EV before matching and merging. Peng et al. then applied another CNN to simultaneously fix alignment errors and conduct HDR merging. Prabhakar et al.’s method proposes that exposure-invariant features are generated, instead of matching images to a set EV. This enables the number of input LDR images to be scalable and is generally effective; however, results suffer greatly when few images are used. Additionally, the images generated by this method tend to suffer in saturated regions, producing less accurate results that fail to mitigate ghosting artefacts in scenes with very high motion.

### 2.4. Transformer Methods

The advent of transformer networks presented new opportunities for researchers to improve HDR performance. Qu et al. [12] proposed the use of a transformer network to create a self-supervising HDR network that is not reliant on training via ground truth images. They combined a CNN module with a transformer module to both exploit global features via the CNN and local features via the attention mechanics inherent to transformers. Their network performs three tasks: learning scene content as well as luminance, learning texture as well as detail information, and learning structural information. These three tasks assist the network in being able to generate natural HDR images that are less noisy and more consistent than other competitive offerings. However, the network is expensive and is designed for operation on discrete, 256 × 256 grey-scale images.

### 2.5. Deep Learning

Advancements in computer vision and deep learning research have facilitated an increased interest in single-image HDR, where an HDR image is reconstructed using only a single-input LDR image. The greatest advantage this method possesses over the conventional bracketing/fusion approach is the elimination of alignment issues, as there are no images to align together. However, challenges arise in reconstructing detail from areas of the LDR image that are saturated or under-exposed. This process was greatly enhanced by CNNs, with early works such as Eilertsen et al.’s HDR-CNN [13] focusing on recreating details lost to over-exposure with a fixed iCRF. This limited the flexibility of the method, as the CRF would have to either be assumed or estimated.

Taking a different approach, Zhang et al. estimate under- and over-exposed illumination maps from a single image in their Dual Illumination Estimation method [14]. This provides a set of psuedo multi-exposure fusion images to conduct multiple exposure fusion on, producing an HDR result. Whilst their method is computationally simple, cheap and effective, it struggles to suppress noise, resulting in an image that is significantly noisier than competing solutions. Additionally, it fails to resolve detail in areas of extreme over-/under-saturation.

Chen et al. proposed an SHDR that employs generative adversarial networks (GANs) to improve performance. Their SHDRI-GAN method [15] implements two GANs: First, an exposure calibration GAN (VET-GAN), which generates an over-/under-exposed right/left view from inputted left and right view images. Second, their HDR fusion GAN fuses the over- and under-exposed image pair from either the left or right view, and then estimates detail that would be present in a three-image fusion. The VET-GAN provides a more accurate replacement to a series of complex operations such as stereo matching, warping and hole filling, whilst the dual fusion and estimation process reconstructs more detail from an image pair than would otherwise be feasible. Their HDR Fusion GAN, however, relies on a fixed gamma correction parameter for initial estimation of the HDR image, limiting the accuracy and flexibility of their solution.

**Figure 1:**
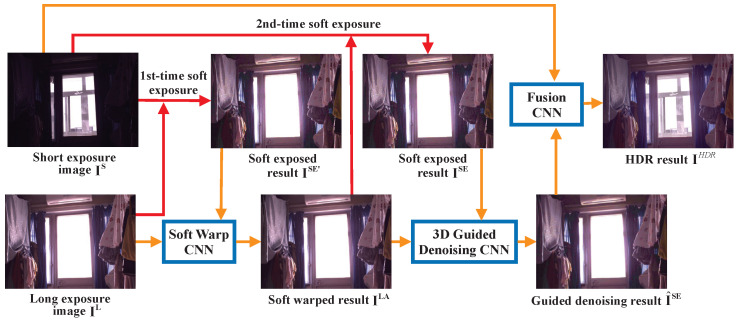
Dong et al.’s MIEHDR pipeline [16].

Instead of weighting each stereo image equally, some methods opt to treat one image as a ‘reference’, which guides matching and enhancement of a complementary image. This can simplify or improve aspects of the stereo HDR process, which Dong et al.’s Main Image Enhancement HDR (MIEHDR) [16] method (see Figure 1) exploits by enhancing the dynamic range and detail of a main query image via a longer exposure reference image. The authors use soft exposure via histogram equalisation to simulate a longer exposure on the under-exposed complementary image. Following the soft exposure are three neural networks that conduct warping, denoising and fusion on the input images. The authors’ technique is able to recreate the HDR image without obvious artefacting or noise. However, the method fails to account for all camera asymmetry in smartphone cameras (e.g., FoV, aperture or resolution). This is exhibited in their use of homogenised camera systems to capture LDR images for input.

### 2.6. Mixed-Focal-Length Methods

**Figure 2:**
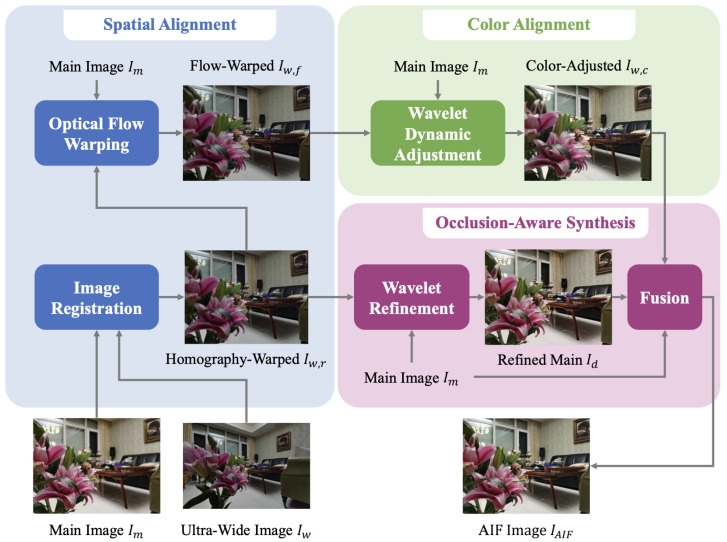
Luo et al.’s asymmetric photo-synthesis pipeline [6].

Due to the fixed aperture of smartphone cameras, research has been conducted into synthesising an evenly focused image (i.e., without a depth of field effect) via fusing images with differing focal lengths. Luo et al. [6] achieved focus synthesis via fusing an image from ultra-wide and wide-angle input images (see Figure 2). Significant challenges are present in aligning asymmetrical images under different parameters and ISP pipelines. To align images, a homography and flow-warping process was applied, as a single homography warping was impossible due to larger background disparities in comparison to the foreground. Hence, key points were extracted and processed to compute initial correspondences between the images, then pruned using a neural network. The ultra-wide-angle image was then warped to provide an approximately aligned image. To refine the alignment, a flow map was estimated and aligned. A series of wavelet transformations and CNNs align the ultra-wide colour profile to that of the reference wide-angle image. Finally, the images were fused using a CNN. Their overall alignment process could fail under certain conditions, showing warping artefacts; however, this appeared to have little impact on the qualitative and quantitative data they produced, likely due to their fusion CNN.

### 2.7. Contributions of This Work

The above evaluation of previous works leads to the identification of three clear gaps in current methods:

1. No implementation exists for aligning differently exposed images from asymmetric lenses.
2. No implementation exists for taking under-/over-exposed LDR images from asymmetric lenses and fusing these into an HDR image.
3. Existing implementations for generating HDR images from multiple LDR images struggle with occlusions and temporal stability.

This work tackles these challenges by developing a new pipeline for asymmetric stereo high dynamic range imaging (ASHDRI). The key contributions of this work are as follows:

1. A pipeline capable of taking differently exposed LDR images from asymmetric lenses and aligning them in preparation for HDR fusion.
2. For the first time, showing a pipeline capable of fusing these aligned LDR images into a single HDR image, capable of gracefully dealing with occlusions and temporal instability.

## 3. Methodology

**Figure 3:**
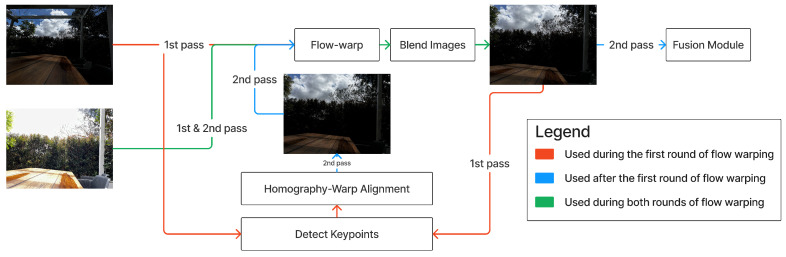
Defined ASHDRI process.

To achieve single-shot ASHDRI, a three-module process was defined (see Figure 3).

1. **Exposure Equalisation Module**—where input images will be aligned to a similar exposure level.
2. **Warp-Alignment Module**—where equalised images are warped and aligned to a similar plane.
3. **HDR Fusion Module**—where aligned images are fused to produce an HDR result.

The following subsections describe how the solution was implemented, how results were gathered, a discussion of evaluation metrics and degradation measures, and a discussion on qualitative testing.

### 3.1. Solution Implementation

This solution was inspired by Luo et al.’s work in asymmetric focus synthesis [6] and Dong et al.’s MIEHDR pipeline [16]. However, the difficulty of the problem necessitated various innovations.

#### 3.1.1. Exposure Equalisation

As the warp-alignment module relies on a high degree of photometric consistency between images, images need to be matched to a similar exposure level. To achieve this, histogram equalisation was implemented for both input images. Histogram equalisation was chosen as it was cheap, reliable and performed sufficiently well.

#### 3.1.2. Warp Alignment

The accumulation of occlusion and perspective errors necessitates expensive operations for the warp-alignment module. A five step process was defined to ensure the alignment is accurate, robust and reliable.

1. Conduct flow-warping on the under-exposed ultra-wide image using the over-exposed wide-angle image as reference.
2. Conduct a confidence map-informed blending operation to minimise flow-warping errors.
3. Detect affine shape/keypoint pairs between the ultra-wide image and the homography-warped ultra-wide image.
4. Use keypoints to warp the ultra-wide image via a homography transform.
5. Repeat steps 1–2 with the ultra-wide image replaced with the affine-warped ultra-wide image.

**Figure 4:**
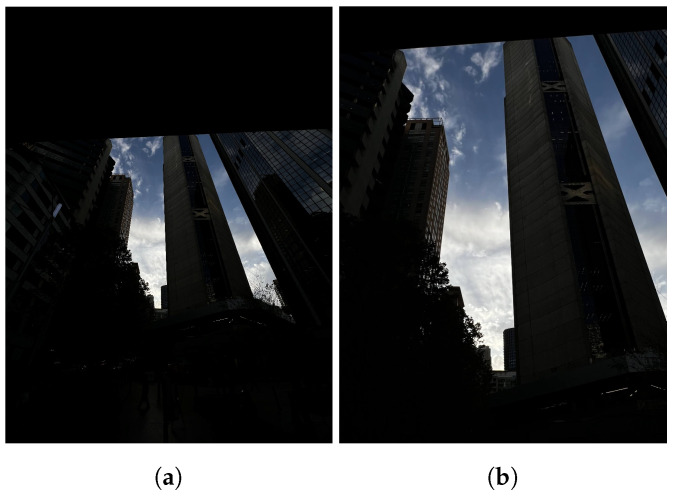
Homography-warping process. (**a**) Input ultra-wide. (**b**) After homography warping.

Mishkin et al.’s Affnet model [17] was used to detect affine shape and keypoint pairs. The model’s strength in matching local affine-covariant features was a deciding factor. Additionally, Affnet demonstrated excellent performance matching features between image pairs with wide exposure differences. Keypoints were filtered via random sample consensus (RANSAC) and used to calculate a homography matrix, through which the ultra-wide images were warped (see Figure 4) [18].

Differences in scene geometry and parallax errors resulted in post-homography-warping images that still exhibited significant local misalignment artefacts, necessitating a localised warping operator. Flow-warping’s per-pixel level operation ensured it was a strong candidate for this operation. The ill-defined nature of the problem (wide differences in image structure, content and colour profiles due to inherent exposure and scene-view differences) led to poor performance on cheaper flow-warping operators. As such, Truong et al.’s PDC-Net+ (Probabilistic Dense Correspondence Network) [8] was chosen for this task. Not only did the model align images better than competing models, it also did so whilst providing a confidence map for the result, which proved critical for post-processing operations.

To improve the accuracy of the flow-warping operation, a pre-processing filter was applied to the wide-angle reference image. The reference image was colour-matched to the query image (non-warped image in the first pass or homography-warped ultra-wide-angle image for the second pass). This was achieved via two methods: Reinhard et al.’s method [19] for the first-pass and Hahne et al.’s Histogram Matching-Monge-Kantorovich Linearization-Histogram Matching (HM-MKL-HM) method for the second-pass [20]. HM-MKL-HM correlates colour details with a greater degree of accuracy whilst keeping noise outside of saturated areas to a minimum. However, this noise became a significant issue when conducting homography-warping. As such, despite its lower accuracy, Reinhard et al.’s colour-matching method performs better when used in the first pass. HM-MKL-HM was also implemented in the alignment module’s next stage, the blending operator.

**Figure 5:**
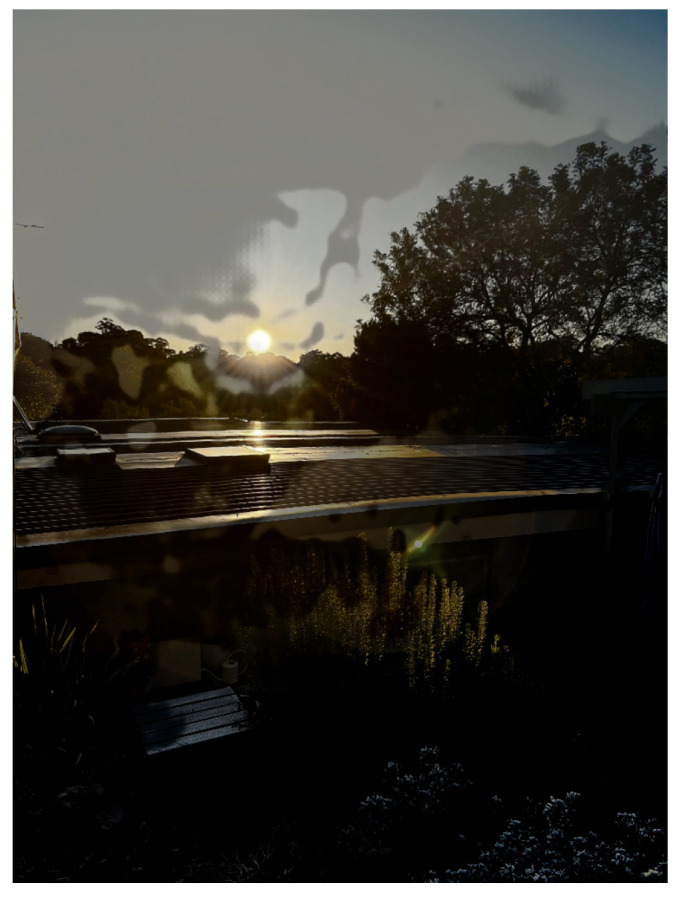
Image post-blending.

PDC-Net+’s confidence map provided a method to alleviate any accumulated errors presented by the flow-warping operation. Pixels with non-confident matches are blended with a colour-matched pixel from the wide-angle ‘reference’ image. Colour and structural details in the blended image are treated differently, as each has distinct effects on the resultant image. Discontinuities in the structure affect the accuracy and subjective appeal of the final blended image. Additionally, preserving as much colour detail as possible from the ultra-wide image reduces noise from colour-matching and contains more relevant data to reconstruct an HDR image. Before blending structural values in the image, the confidence map values are passed through a sigmoid function to smooth out detail reconstruction. An example of an image after blending, and before colour correction, is given in Figure 5.

Colour detail is decided in a binary fashion. When PDC-Net+ is more than 20% confident in its result, the colour detail from the flow-warped frame is chosen; otherwise, the model uses detail from the colour-matched reference frame. The image is converted to a YUV format, where the Y channel (luminance) preserves structural grey-scale detail, whilst the U and V channels (chroma components) contain the colour detail.

**Figure 6:**
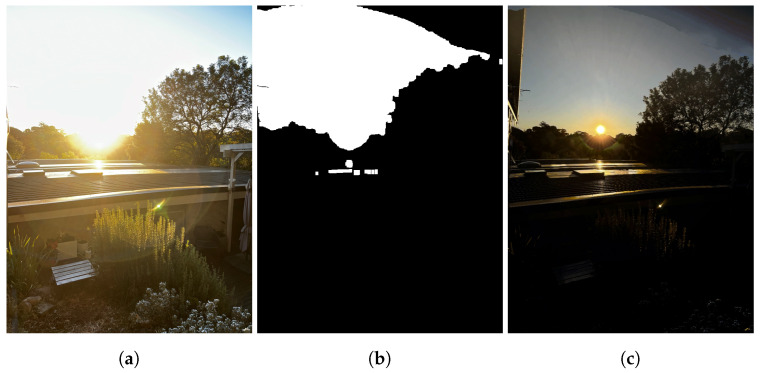
Exposure thresholding process. (**a**) Wide-angle input. (**b**) Exposure mask. (**c**) Post-thresholding image.

As PDC-Net+ struggled to produce confident flow-vectors for areas of significant low-frequency detail, saturated areas in the over-exposed reference image were blended inappropriately. This resulted in an unacceptable loss of detail and unsightly artefacting. To remedy this, a mask identified areas of and around pixels with Y values above 240. As the area is saturated in the reference image, ghosting is not a concern. Hence, these pixels were replaced with their corresponding values in the homography-warped reference image (see Figure 6c). This operation was only performed on the second-pass of the flow-warp, as thresholding can otherwise cause difficulty in detecting reliable keypoints in the homography-warping stage.

**Figure 7:**
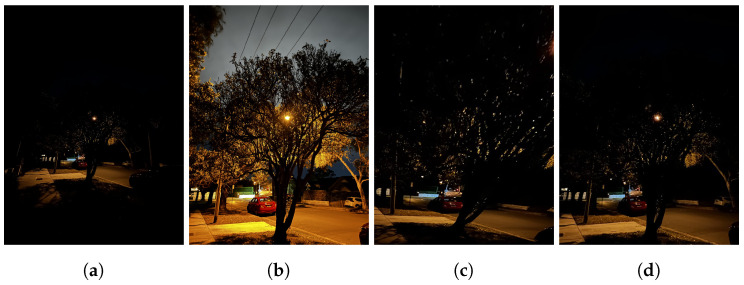
Performance of the homography-warping process under low-light scenarios. (**a**) Ultra-wide-angle input. (**b**) Wide-angle input. (**c**) One-step homography-warping output. (**d**) Two-step homography-warping output.

Various limitations inherent to the flow-warping and affine-shape detection processes necessitated the dual-flow-warping operation. Firstly, over-/under-saturated detail between the ultra-wide/wide image pair led to difficulties in detecting features and, thus, correlating shapes reliably between the two images. As such, in cases of extreme dynamic range, or poorly lit scenes, an accurate homography matrix could not be generated, resulting in a poorly aligned frame (see Figure 7c). Errors in the homography warp would propagate and reduce the quality of the final HDR result significantly.

Flow-warping is conducted per-pixel and, as such, discrepancies in the view of a scene (such as those caused by differences in image exposure) do not affect the alignment as globally as they might in the whole-image homography-warping case. This ensures it is uniquely suited for aligning the differently exposed images. However, its high complexity and memory cost necessitate reductions in the input resolutions of images to approximately 720 p. When warping the ultra-wide case, the image is effectively cropped into a half-size image, resulting in effective resolutions of 360 p for ultra-wide images. This was deemed unacceptable and, as such, a secondary, lower cost warping operation that can operate at a higher resolution (the homography warp) will use this low-resolution flow-warped image as a ‘reference’ to guide a higher-resolution but less-accurate warping of the ultra-wide. As the affine-warp reduces the viewpoint of the ultra-wide to the smaller, lower-resolution, wide-angle equivalent at an already higher input resolution, the flow-warp will not reproduce this ‘cropping’ effect. This preserves enough resolution for a subsequent 720 p flow-warp to not exhibit unacceptably degraded image quality.

#### 3.1.3. HDR Fusion

**Figure 8:**
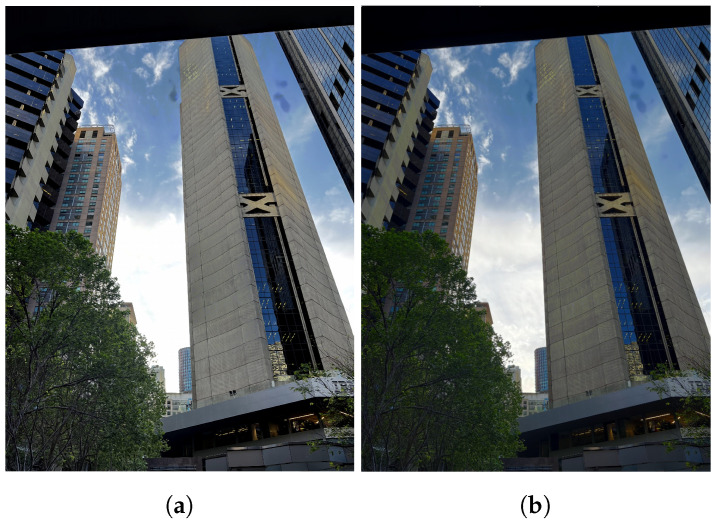
Different HDR outputs. (**a**) Mertens Fusion output. (**b**) TransMEF output. *Note how TransMEF is able to recreate detail in the cloudy sky that is lacking in Mertens Fusion*.

Qu et al.’s TransMEF (multi-exposure fusion) transformer module [12] was chosen for synthesising an HDR image from the warp-aligned query and wide-angle reference images. The model outperformed competing methods when only two inputs were applied, preserving detail to a significantly greater degree than other viable solutions, such as Mertens Fusion (see Figure 8). As the model outputs a grey-scale HDR image, colours were re-applied to the image by treating the grey-scale output as the Luminance (Y) channel of a YCbCr image. Xu’s method [21] maps the blue difference and red difference (Cr and Cb, respectively) components from the two input images.

### 3.2. Gathering Results

Due to the novelty inherent in ASHDRI, no existing dataset is capable of sufficiently assessing the performance of the proposed method. As such, images were captured using two phones in distinct scenes with differing lighting conditions and obstacles that could cause difficulty for HDR generation. This included harsh lighting that occluded scenery from one or both views and scenery that existed in only one viewpoint. Images were captured on an iPhone 13 Pro and a Samsung Galaxy S21+. On the iPhone, images were captured using Ashutosh Billa’s ProCamera—Capture RAW photos application [22], as it automatically captured image triplets with exposure values of −2, 0 and 2 EV. This ensured that the proposed method could be compared against existing methods fairly by generating HDR images from the ultra-wide/wide image pair at distinct EVs, and from a wide/wide or ultra-wide/ultra-wide image pair. Results were processed on a Windows 10 computer with 32 GB of DDR4 RAM, an AMD Ryzen 3600X CPU and an NVidia RTX 3070 GPU (sourced in Sydney, Australia).

### 3.3. Comparing to Existing Solutions

The novelty of the work and the potentially wide variance in applications of ASHDRI necessitated a comprehensive testing suite to compare the algorithm against existing solutions in a variety of contexts. ASHDRI results were compared to single-view (Dual illumination estimation [14]) and multi-view (FMMEF [23], Ghost-Free MEF [24], PASMEF [25], TransMEF [12] and Mertens [26]) HDR solutions (unfortunately, an open-access repository for SHDRI-GAN [15] was not available) in three separate contexts:

1. MEF with an unwarped ultra-wide image and a wide-angle image.
2. MEF with two misaligned wide-angle images (to simulate motion).
3. MEF with two aligned wide-angle images.

In every case, single-image HDR solutions were generated from the middle-exposed (0 EV) frame from the wide-angle camera. Comparing against this variety of scenarios ensured that results could establish a clearer picture of where the proposed pipeline excels in comparison to existing solutions, and areas of potential improvements. The potential improvements for this work are discussed in more detail in Section 5.2.

### 3.4. Evaluation Metrics

The complexity in the approach taken by this paper necessitated a variety of measurement metrics to objectively assess the performance in a variety of scenarios. As an objective ground truth was not available (due to the lack of access to HDR equipment), a multi-exposure Mertens fusion from three wide-angle aligned images was used as a substitute.

However, when measuring misaligned frames, the generated ground truth also inherits misalignment artefacts. This results in a blurry image that poorly represents the underlying scene. As such, instead of being compared against a ground truth, competing solutions will be compared directly against the proposed pipeline when considering misaligned frames. A no-reference HDR-VDP pre-trained model will also be used as another indicator.

### 3.5. Degradation Measurements

To measure the degradation of the image relative to an input ground truth, peak signal-to-noise ratio (PSNR) and structural similarity index measure (SSIM) were used.

#### 3.5.1. High-Dynamic-Range Visual-Difference-Predictor 3

The Just Objectionable Difference score from the High-Dynamic-Range Visual-Difference-Predictor 3 (HDR-VDP-3) [27] was used to indicate the subjective ‘quality’ of the HDR generation relative to a ground-truth (the ‘Q’ score).

#### 3.5.2. NoR-VDP-Net

As the testing methodology involves assessing HDR generation across misaligned frames, a ground truth image is not necessarily always available. In these scenarios, Banterle et al.’s no reference implementation of HDR-VDP [28] provides a suitable alternative. The metric has been trained on HDR-VDP and, as such, is able to predict what the quality of the generated HDR might be.

### 3.6. Qualitative Testing

Discrepancies in tone-mapping, the ability to deal with misalignment in objects and other functionalities present in different HDR methods were difficult to assess fairly through quantitative methods. As such, images were subjectively analysed in addition to the objective performance metrics already established. Particular focus was given to different methods’ ability to resolve ghosting artefacts, the presence of mid-range detail and the subjective ‘appeal’ of the resultant HDR image.

## 4. Results and Discussion

The model demonstrated good performance across a variety of scenes and lighting conditions, with significant detail recovered from harsh lighting conditions. However, the image alignment degrades significantly in dynamic low-light scenarios.

### 4.1. Warping

Despite demonstrating promising results in relatively evenly lit scenes, the warping method has variable levels of success for more difficult lighting scenarios. However, the backup blending methods employed by this paper ensure that ghosting and noise are minimised whilst as much detail from the ultra-wide frame is preserved as possible.

#### 4.1.1. Affine Warp

**Figure 9:**
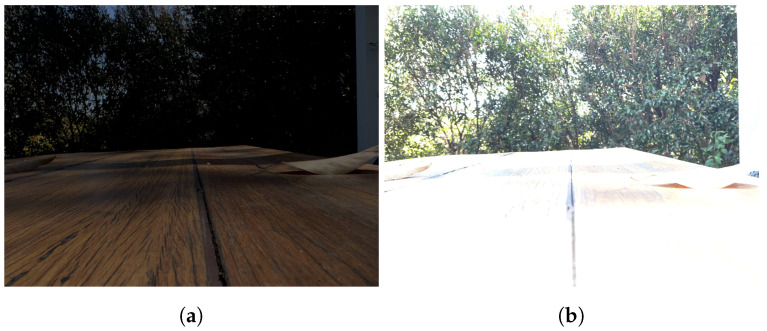
Misalignment on the second-pass homography warp due to saturation in the +2 EV image. Note the position of the leaf on the table. (**a**) Warped query image. (**b**) Reference image.

The affine-warping process is effective for a variety of lighting conditions; however, when faced with dynamic, over-/under-saturated scenarios, it fails to properly align frames (see Figure 7c and Figure 9a). This propagates into the flow-warping and blending operations. Poor alignment likely occurs as there is insufficient detail to extract features from the entire image. As such, keypoints are only detected in a small region of the image, reducing the overall reliability of the warping operation.

#### 4.1.2. Flow-Warp

Flow-warping, when combined with the post-process blending and thresholding operators, performs effectively and robustly in a variety of lighting scenarios. In evenly lit scenes, the model is able to match and warp images with high confidence. Regions of poor confidence are remedied by the colour-matching/blending operators, resulting in a consistent, well-resolved image.

However, the model struggles to resolve areas of differently exposed, low-frequency detail. This is likely due to the lack of a structure to compare regions against, resulting in low confidence for the entire region. Whilst this is somewhat remedied by the blending operators, they cannot fully account for under-exposed, low-frequency areas, resulting in an image that does not return as much detail as desired.

The blending and thresholding operators minimise the impact of misalignment artefacts. The HM-MKL-HM colour-matching operator is able to align the colour profiles of the two disparate images effectively, albeit with a large degree of noise, which is inevitable because histogram methods operate over the entire image. Saturated regions exhibit the highest degree of noise; however, this is addressed by the thresholding operator.

**Figure 10:**
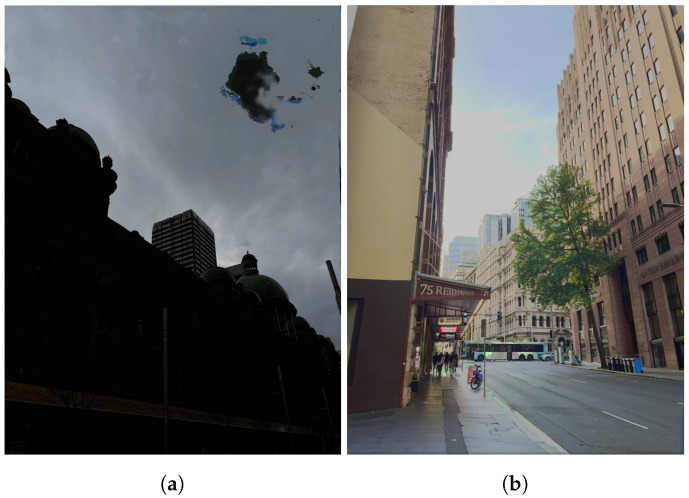
Noise from the colour-matching process that has not been properly blended. (**a**) Colour-mismatch noise. (**b**) Green discolouration artefacts.

The lack of sophistication in the thresholding process causes issues. The arbitrary 240 luminance cutoff in conjunction with the square kernel result in a process that can exhibit unsightly artefacts (see Figure 10). Figure 10a demonstrates artefacting due to low-frequency detail below the 240 luminance threshold. The pixelated appearance of the shape is a consequence of the square kernel of the thresholding operator. Figure 10b demonstrates increased noise due to over-exposed detail and misalignment between frames, resulting in the presence of unnatural greens on the bus and footpath. Future work could remedy this by integrating the process into a neural network for more nuance or investigating other colour-matching methods.

### 4.2. HDR Generation

#### 4.2.1. Ultra-Wide/Wide Fusion

**Figure 11:**
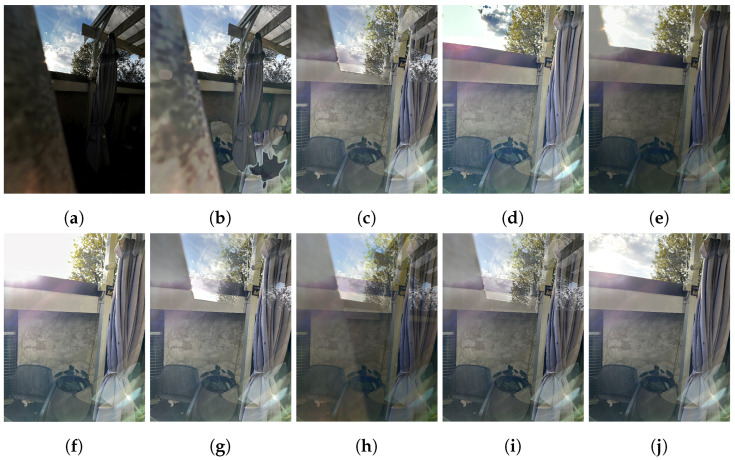
Comparison of methods when using the same inputs as the proposed pipeline (−2 EV ultra-wide, +2 EV wide-angle image). (**a**) Input −2 EV. (**b**) FMMEF. (**c**) Ghost Free MEF. (**d**) Dual Illumination Estimation. (**e**) Our result. (**f**) Input +2 EV. (**g**) PASMEF. (**h**) TransMEF. (**i**) Ultra-wide/wide Mertens Fusion. (**j**) Ground Truth Mertens.

As demonstrated in Figure 11, the proposed pipeline outperforms all compared algorithms when considering ultra-wide and wide-angle image pairs. The proposed method is able to capture detail from the two frames more evenly, whilst avoiding the ghosting present in all the compared solutions. Other HDR solutions are unable to resolve misalignment issues and the significant aperture and ISP differences, meaning their output resembles two overlaid images rather than a single cohesive scene.

**Figure 12:**
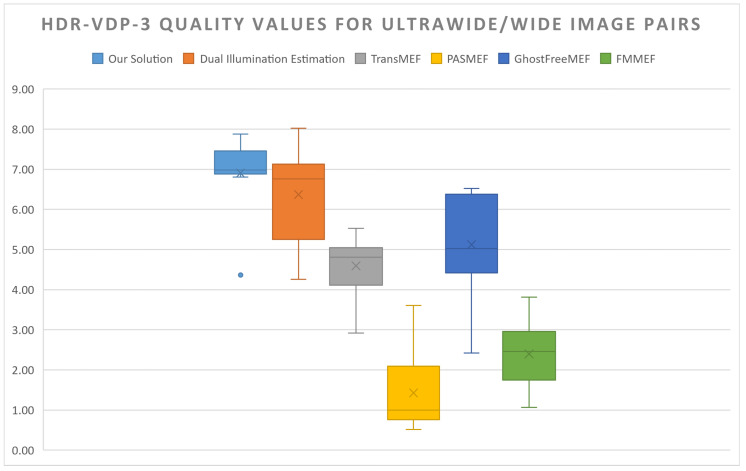
Comparisons between competing solutions in which they have ultra-wide/wide-angle input pairs. From left to right: ASHDRI, Dual Illumination Estimation, TransMEF, PASMEF, GhostFreeMEF, FMMEF.

Other methods have significantly lower HDR-VDP-3 Q scores, with the pipeline performing on average 1.5× better than compared methods (see Figure 12). Only the single-image, psuedo MEF method of dual illumination estimation is able to remain competitive with ASHDRI results, being the only solution to be within 10% of ASHDRI’s quality scores on average.

#### 4.2.2. Misaligned Fusion

**Figure 13:**
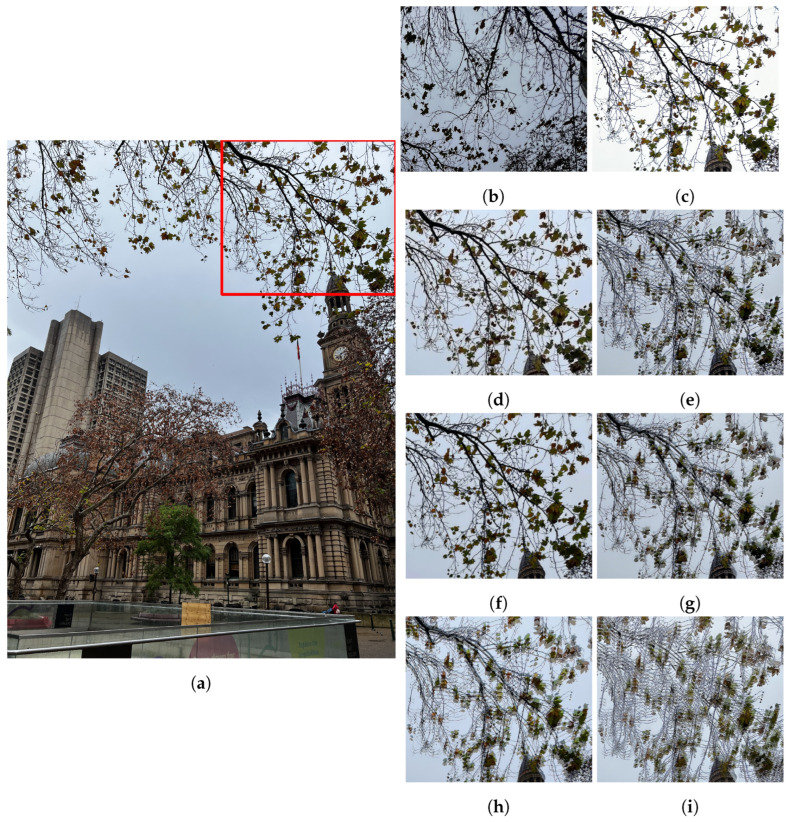
Comparison of misaligned MEF methods. The red box shows the location of the zoomed in images. (**a**) 0 EV reference image. (**b**) Input −2 EV. (**c**) Input +2 EV. (**d**) FMMEF. (**e**) Ghost Free MEF. (**f**) Our result. (**g**) PASMEF. (**h**) TransMEF. (**i**) Mertens Fusion.

When compared to misaligned wide-angle images, the proposed pipeline still outperforms competing solutions (see Figure 13, where most competing solutions exhibit ghosting in the trees and rail-guard). Whilst the misalignment artefacts from competing solutions are lessened in comparison to ultra-wide/wide input pairs, they are still significant. Some solutions fare better in resolving the scene differences; however, none resolve the large scene differences as effectively as the proposed pipeline. This is likely a combination of large alignment differences as well as a lack of input images to properly resolve a single view for a scene. The significant ghosting of competing solutions results in an image that is qualitatively and quantitatively worse than the proposed method.

Solutions that take a more considered approach to misalignment artefacts, such as Li et al.’s FMMEF [23], are able to resolve some of the misalignment artefacts (see Figure 13d). However, resultant images can still exhibit significant noise or misalignment in difficult scenarios (such as in Figure 11b) that the proposed solution can resolve. This difficulty in aligning images is reflected in their poor HDR-VDP-3 scores; however, a more nuanced interpretation is required when analysing the quantitative results returned in a misaligned scenario.

**Figure 14:**
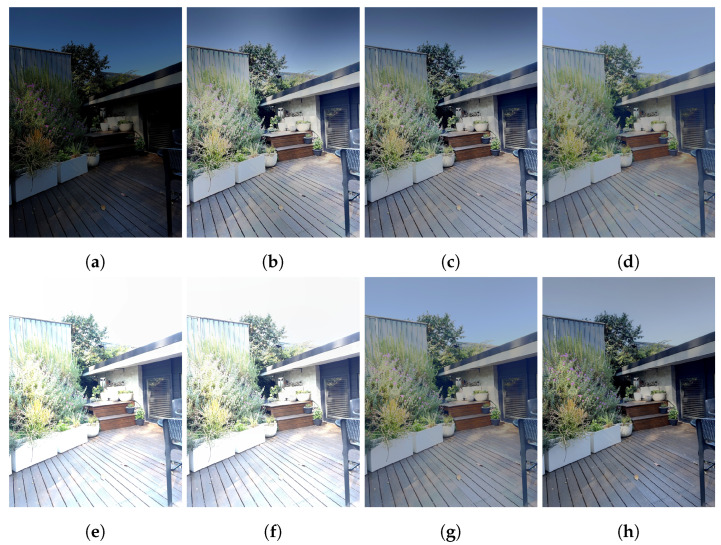
Comparison of aligned Multi-Exposure Fusion methods. (**a**) Input −2 EV. (**b**) FMMEF. (**c**) Ghost Free MEF. (**d**) ASHDRI Result. (**e**) Input +2 EV. (**f**) PASMEF. (**g**) TransMEF. (**h**) Ground Truth Mertens.

**Figure 15:**
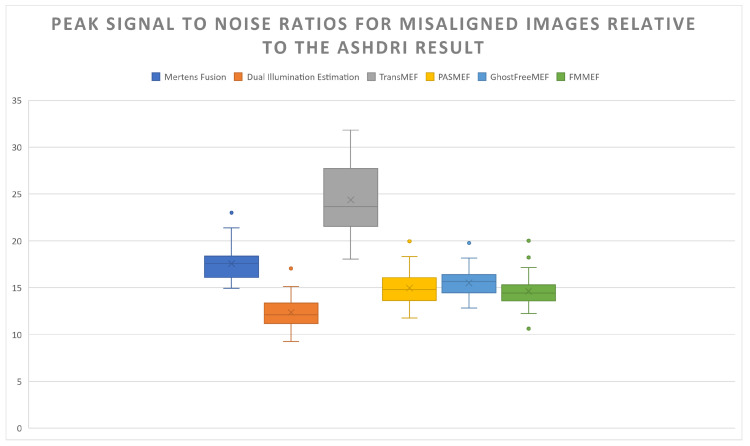
Noise comparisons against competing solutions in which they have ultra-wide/wide-angle input pairs. From left to right: Mertens Fusion, Dual Illumination Estimation, TransMEF, PASMEF, GhostFreeMEF, FMMEF.

SSIM scores indicate that the majority of the competing solutions are only able to recreate an image that is 70% similar to the ASHDRI result. However, the high performance of TransMEF and poor performance of Dual Illumination Estimation demonstrate the weaknesses of this model without a ground truth. As Dual Illumination Estimation reconstructs an HDR image from the medium-exposed input, the output possesses an inherently different structure leading to poor SSIM scores despite the strong subjective performance of the model. The high scores of TransMEF in this context, despite poor subjective results (see Figure 13h), highlight the biases of SSIM due to the reference image (the ASHDRI result) using the same fusion method. As such, the model considers the colour profiles and flaws of the TransMEF model (such as the poor contrast in the plants seen in Figure 14d,g) as the truth of the underlying scene, despite this not being the case. This similarly explains the PSNR scores seen in Figure 15.

**Figure 16:**
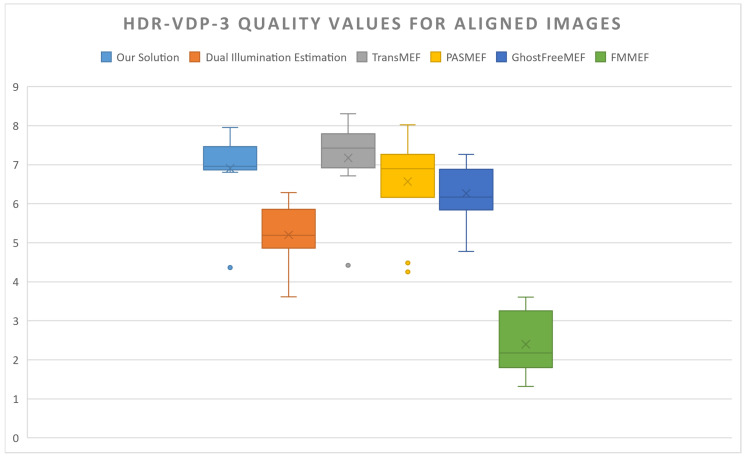
HDR quality comparisons between competing solutions in which they have aligned wide-angle inputs. From left to right: ASHDRI, Dual Illumination Estimation, TransMEF, PASMEF, GhostFreeMEF, FMMEF.

The HDR-VDP-3 results similarly show a bias towards TransMEF and against Dual Illumination Estimation results; however, the results more closely correlate with subjectively observed outputs. Methods that were able to overcome blurring artefacts more effectively, such as PASMEF and FMMEF, have scores that indicate a high level of quality relative to the ASHDRI result. Those that struggle to resolve large-scale misalignment artefacts such as Mertens Fusion and GhostFreeMEF exhibit significant degradation in their performance relative to their aligned results in Figure 16.

**Figure 17:**
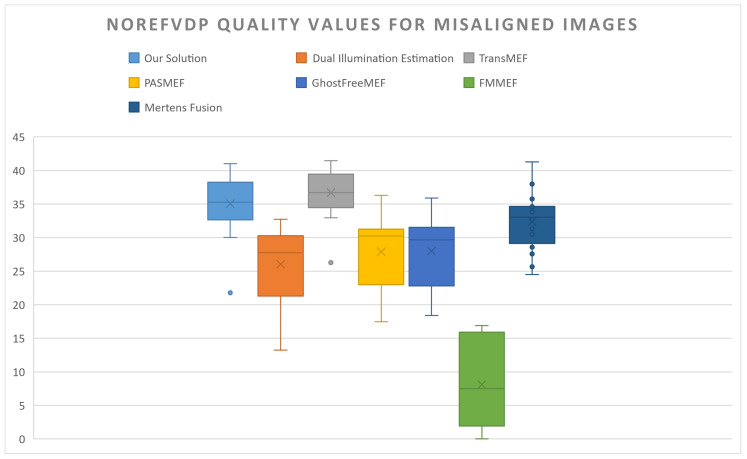
No-reference HDR quality comparisons between competing solutions in which they have misaligned input pairs. From left to right: ASHDRI, Dual Illumination Estimation, TransMEF, PASMEF, GhostFreeMEF, FMMEF. Note: NoRefVDP does not have a maximum quality value, unlike HDR-VDP-3.

The NoRefVDP results (see Figure 17) indicate that the ASHDRI pipeline performs well on misaligned images relative to other solutions; however, this again differs from the qualitative evaluation of the results. NoRefVDP is designed to detect quality results considering already known forms of image degradation. As results were processed with a pre-trained NoRefVDP model, it is unlikely to have been trained on the unique forms of noise and blending artefacts present in the proposed asymmetric HDR use-case and might not be trained to register blurring. This can be seen by the high Q scores for the TransMEF module, despite exhibiting high blurring (see Figure 13h).

#### 4.2.3. Aligned Fusion

Artefacts from the warp-alignment module, as well as scene differences between the ultra-wide and wide-angle viewpoints, cause a degradation in ASHDRI’s performance when competing solutions were given aligned +/−2 EV images as inputs. Despite this, many models face difficulty in generating an image with no medium-exposure image, resulting an output that is biased towards either the over- or under-exposed image. In comparison, ASHDRI is able to resolve an image with more mid-range detail that is lacking in many competing solutions.

**Figure 18:**
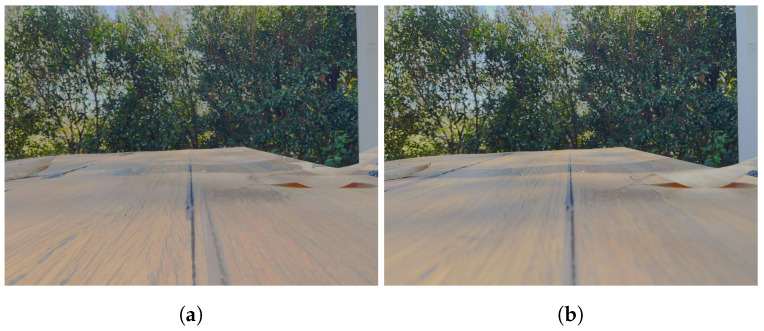
Misalignment propagates to the HDR process, resulting in an image with ghosting artefacts. (**a**) ASHDRI result. (**b**) TransMEF Fusion with aligned wide-angle inputs.

Quantitative results demonstrate that the pipeline still performs well in these more difficult tests. HDR-VDP 3 quality scores are competitive with existing solutions (see Figure 16). However, results returned from the model can exhibit artefacting or unnatural colour profiles in comparison to competing solutions. There are numerous contributing factors to this. Firstly, blending artefacts from the alignment solution contributes to spots of colour mismatching and noise in the resultant HDR solution. Secondly, while Xu’s colour reconstruction algorithm accompanying TransMEF is effective at resolving mid-range detail, it does not always produce natural colour or contrast profiles (see Figure 18).

Comparisons to single-image solutions are mixed, revealing the strengths and weaknesses of the pipeline. The pipeline deals with saturated detail and colour deviation better than single-image solutions, but misalignment artefacts can degrade performance significantly. Figure 11d demonstrates this, where the single-image solutions boost saturation in the sky, resulting in an unnatural and inaccurate, albeit subjectively appealing, appearance. The proposed pipeline, reconstructs more detail in the sky, but also inaccurately, due to the occluding ladder present in the ultra-wide view. Results from single-image HDR solutions can also amplify noise significantly, especially in low-light scenarios. This results in PSNR scores that are typically well below the proposed pipeline. Single-image solutions perform poorly in SSIM, PSNR and HDR-VDP-3 metrics, which is likely a consequence of the increased noise or saturated detail in the middle-exposed frame that cannot be recreated by two-image solutions.

As the robust alignment module is able to create an image that correlates strongly with an under-exposed wide-angle input, the HDR performance relative to the same TransMEF model with a +/−2 EV wide-angle input pair is very similar. Most results exhibit HDR-VDP-3 quality scores within 10% of TransMEF with two wide-angle inputs.

However, when the alignment module fails, there is a significant degradation in the performance of the pipeline (see Figure 18). This can be caused by a combination of poor alignment in the homography warp combined with the blend/thresholding not being able to consider all poorly aligned detail (i.e., edges of over-exposed regions), resulting in a visibly poor blending process. The base TransMEF model performs on average 4% better than ASHDRI, another indicator of the pipeline’s degradations. More edge cases such as this could exist, and future work could investigate methods to mitigate their effects, such as a better colour alignment module.

### 4.3. Performance

The combination of VRAM-intensive flow-warping and the performance-intensive TransMEF module creates a pipeline that is slower than desirable. As seen in Table 1, the alignment and fusion take, on average, 36 s, with all results falling within a 29–46 s range. This is higher than comparative DL HDR solutions, which is likely a consequence of the need for an intensive alignment module. The high performance and video memory requirements of the pipeline mean that it is neither possible to run it in real-time nor for it to operate locally on a smartphone.

The most performance-intensive component of the pipeline is the TransMEF module, which takes approximately 14 s to run. This is likely a consequence of the original model being designed to be run on resolutions of 256 × 256 input images, whilst the test images used an upsampled ultra-wide image and a wide-angle image with resolutions of 3042 × 4032. Therefore, TransMEF processes 192 256 × 256 patches, each taking 0.02–0.1 s. More investigation could be conducted into combining the disparate components of the warp/alignment module into a single network to improve performance and effectiveness. Additionally, the thresholding and blending operators could operate on lower resolution inputs. Future work could focus on designing and training an optimised end-to-end transformer network for the express purpose of ASHDRI.

## 5. Conclusions and Future Work

### 5.1. Conclusions

Asymmetric Stereo High Dynamic Range Imaging has been achieved via a three-module solution: exposure equalisation, warp alignment and HDR fusion. Exposure equalisation aligns images to a similar exposure level through histogram equalisation. The warp-alignment module is an interconnected series of deep learning methods to match and align images through homography and flow-warping. The module also uses blending and thresholding operators to remedy issues caused by poor warping. This process is repeated twice, with the second pass replacing the ultra-wide image with the output image from the first pass. Finally, Qu et al.’s TransMEF [12] module fuses the images into a single output image.

Results exhibit 1.5× higher HDR-VDP-3 scores on average (indicating that HDR fusion is more visually appealing than competitors) with significantly less misalignment than competing solutions whilst maintaining a similarly accurate recreation of the scene. However, the total time taken and the resources required for the pipeline mean that it is not suitable for deployment to mobile devices.

### 5.2. Future Work

As the model exhibits far stronger performance relative to other solutions in misaligned scenarios, the feasibility of an ASHDRI pipeline is evident. However, there are limitations in the methodology and construction of the pipeline that future work could address. Flaws in the proposed ASHDRI pipeline (e.g., frame and colour misalignment) could be circumvented via the creation of a bespoke ASHDRI convolutional neural network (CNN). Similar to Wang et al.’s reference-based super-resolution pipeline [4], extracting, aligning and fusing images at the deep feature level could improve accuracy, robustness and performance.

The high VRAM requirements and long run-times led to sub-optimal performance that was unviable for local mobile use. Hence, optimisations such as lowering the resolution of alignment and predicting an up-scaled flow-field, or training a model under lower resolution weights, could accommodate lower-end hardware better.

Result gathering and assessment faced a variety of limitations and shortcomings that could be improved in future works. Future work could focus on establishing a larger dataset with a high-quality ground truth in all scenarios using an HDR camera. Higher-quality quantitative assessment, including of motion blur and no-reference HDR, could also be an avenue for future ASHDRI research.

Expanding the ASHDRI process to include input from a telephoto lens is a natural avenue for future work to explore. By treating the wide-angle as a medium-exposure image, keypoint matching will perform better and less mid-range detail will require hallucination. Fusing a colour-matched wide-angle frame with the telephoto (using concepts exploited by Lee et al.’s super resolution pipeline [5]) could account for the lost detail outside the telephoto’s frame. 

## Figures and Tables

**Table 1:** Operating times for sections of the pipeline. All values are seconds. The results are based on a test dataset of 30 images.

Statistic	Flow-Warp	Warp Alignment	Fusion	Total Time
Mean	8.491	12.54	18.16	35.89
Standard Deviation	1.634	1.660	2.599	3.761

## Data Availability

Code is openly available on GitHub: https://github.com/Frussell556/ASHDRI. Images used are not provided due to privacy concerns.
